# Proteomic and Functional Analyses Reveal MAPK1 Regulates Milk Protein Synthesis

**DOI:** 10.3390/molecules18010263

**Published:** 2012-12-27

**Authors:** Li-Min Lu, Qing-Zhang Li, Jian-Guo Huang, Xue-Jun Gao

**Affiliations:** Key laboratory of Dairy Science of Education Ministry, Northeast Agricultural University, Harbin 150030, Heilongjiang, China; E-Mails: liminlu@yahoo.com.cn (L.-M.L.); qzli@neau.edu.cn (Q.-Z.L.); 13946178649@139.com (J.-G.H.)

**Keywords:** proteomic, L-lysine, dairy cow mammary epithelial cells, MAPK1, Stat5, mTOR

## Abstract

L-Lysine (L-Lys) is an essential amino acid that plays fundamental roles in protein synthesis. Many nuclear phosphorylated proteins such as Stat5 and mTOR regulate milk protein synthesis. However, the details of milk protein synthesis control at the transcript and translational levels are not well known. In this current study, a two-dimensional gel electrophoresis (2-DE)/MS-based proteomic technology was used to identify phosphoproteins responsible for milk protein synthesis in dairy cow mammary epithelial cells (DCMECs). The effect of L-Lys on DCMECs was analyzed by CASY technology and reversed phase high performance liquid chromatography (RP-HPLC). The results showed that cell proliferation ability and β-casein expression were enhanced in DCMECs treated with L-Lys. By phosphoproteomics analysis, six proteins, including MAPK1, were identified up-expressed in DCMECs treated with 1.2 mM L-Lys for 24 h, and were verified by quantitative real-time PCR (qRT-PCR) and western blot. Overexpression and siRNA inhibition of MAPK1 experiments showed that MAPK1 upregulated milk protein synthesis through Stat5 and mTOR pathway. These findings that MAPK1 involves in regulation of milk synthesis shed new insights for understanding the mechanisms of milk protein synthesis.

## 1. Introduction

In eukaryotes, protein phosphorylation is among the most important regulatory events in cells, guiding primary biological processes, such as cell division, growth, migration, differentiation, and protein synthesis. Phosphoproteomics has emerged as a powerful technique for the quantitative and qualitative mapping of the whole phosphoproteins in response to many extracellular stimulis [1,2].

Amino acids (AA) are known as anabolic factors, which induce protein gain by stimulating protein synthesis while inhibiting proteolysis. Lysine (Lys) is often the first limiting AA for dairy cows. Several authors have shown that Lys supplementation, at levels above the requirement for maximal growth rate, results in specific and significant effects on body composition [3]. Burgos* et al.* demonstrated that Lys modulates mammary protein synthesis [4].

Although there are some reports about the regulation mechanism of milk synthesis [5,6], the details of the mechanism remain largely unknown. We still do not know the details of the cell signal transduction pathway for milk protein synthesis, including what molecules participate in the Jak2-Stat5 pathway to regulate milk protein synthesis at the transcriptional level, and what molecules affect the mTOR pathway to regulate milk protein synthesis at the translational level, and what molecules transfer the signals of amino acids to Jak2-Stat5 pathway and mTOR pathway. In this study, we used two-dimensional gel electrophoresis (2-DE) to profile the nuclear phosphoproteomics of DCMECs treated with L-Lys in order to identify differentially expressed phosphoproteins. The aim of this paper was to identify novel phosphoproteins that might be related to milk protein synthesis in DCMECs. This will also facilitate a better understanding of the lactation molecular mechanism

## 2. Results and Discussion

### 2.1. Lys Promoted Cell Growth and Increased β-Casein Expression

**Figure 1:**
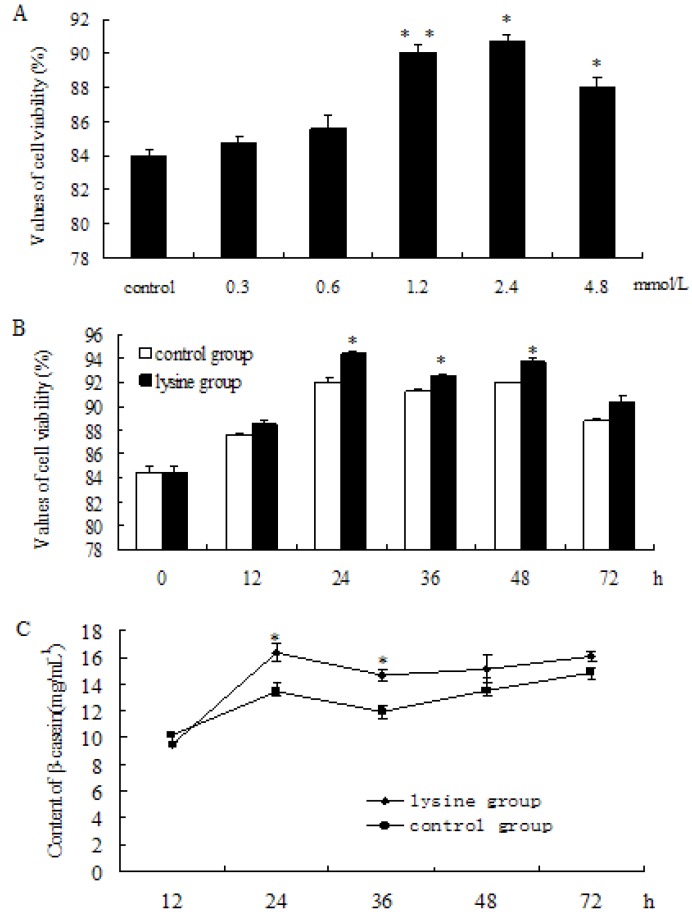
Detection of cell viability and β-caseinsecretion of DCMECstreated with L-Lys. (**A**) Viability of cells at 24 h after adding L-Lys (0, 0.3, 0.6, 1.2, 2.4, 4.8 mmol L^−1^); (**B**) Viability of cells at 0, 12, 24, 36, 48, 72 h after adding L-Lys1.2 mmol L^−1^; (**C**) Changes of β-casein content of DCMECs at 12, 24, 36, 48, 72 h after adding L-Lys1.2 mmol L^−1^. Values are means ± SE (n = 3 per group, biological replicates). * and ** indicate significant differences from values obtained in the control group at the same time point at levels of *p* < 0.05 and *p* < 0.01, respectively.

With the increases of Lys concentration, cell viability in the Lys group was relatively higher, compared to the control group. The results showed that treatment with Lys for 24 h resulted in a dose-dependent increase in cell viability (Figure 1A was significantly increased at 1.2 mmol L^−1^ (Figure 1A, *p* < 0.01), 2.4 mmol L^−1^ (Figure 1A, *p* < 0.05), and 3.0 mmol L^−1^ (Figure 1A, *p* < 0.05); yet, no significant increase was observed at 0.3 mmol L^−1^ and 0.6 mmol L^−1^. To evaluate the effect at different time points, cells were stimulated with L-Lys (1.2 mmol L^−1^), harvested after treatments at 0 h, 12 h, 24 h, 36 h, 48 h, and 72 h, respectively. The results showed that, in comparison to the control group, cell viability significantly increased at 24 h, 36 h, and 48 h (Figure 1B, *p* < 0.05). According to β-casein standard curve analysis, the contents of β-casein significantly increased at 24 h (*p* < 0.05) and 36 h (*p* < 0.05), compared to the control group (Figure 1C). The results suggested that Lys increased β-casein secretion of DCMECs.

### 2.2. Lys Caused Up-Regulated Expressed Phosphoproteins

**Figure 2:**
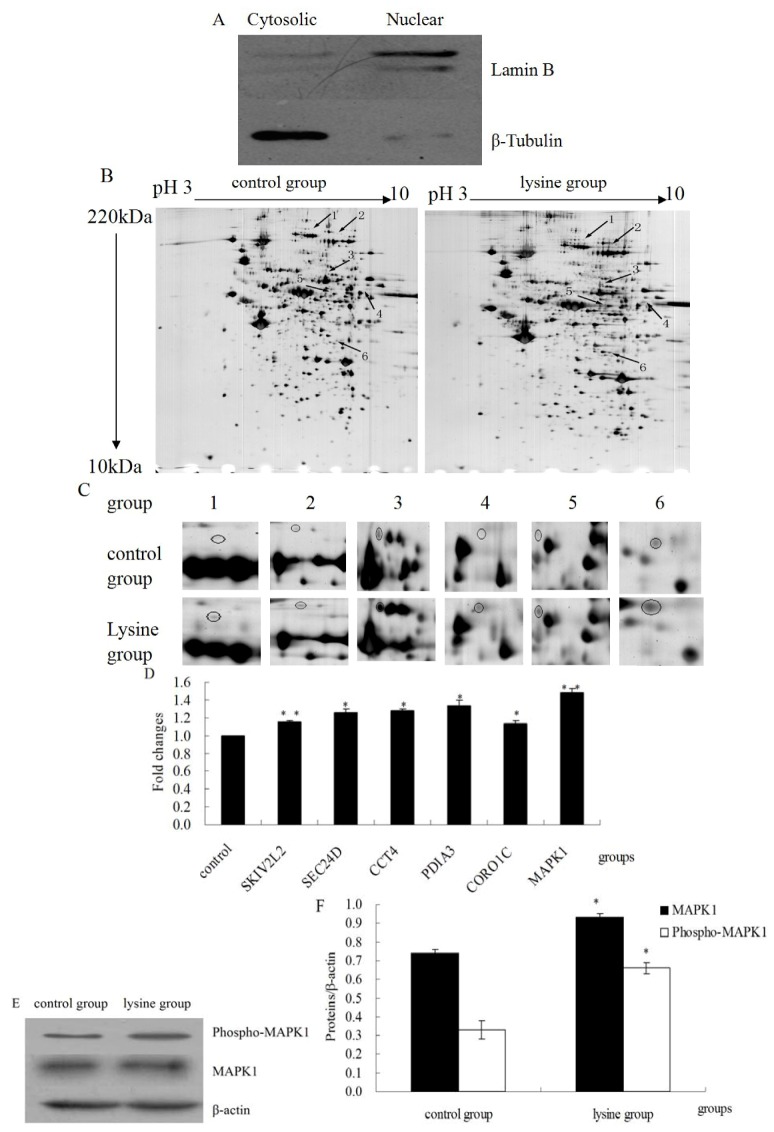
The nuclear phosphoproteomics of DCMECs treated with L-Lys. (**A**) Western blotting analysis showing the expression of markers in nuclear (Lamin B) and cytosolic (β-Tubulin) extracts of DCMECs; (**B**) Comparison of the gels of the control group with those of L-Lys-treated group. The differentially expressed proteins are indicated with arrows and numbered; (**C**) Close-up of the six differentially expressed proteins; (**D**) Comparative expression of genes as determined by RT-PCR. β-actin as control under the same condition. The ratio of the control group was regard as one; (**E**) Western blotting results of phospho-MAPK1, MAPK1 and β-actin; (**F**) Results of gray scale scan of phospho-MAPK1 and MAPK1.β-actin as control under the same condition. Values are means ± SE (n = 3 per group, biological replicates). * and ** indicate significant differences from values obtained in the control group at levels of *p* < 0.05 and *p* < 0.01, respectively.

Nuclear proteins were extracted as described in the Experimental section. Protein expression of cytosolic (β-tubulin) and nuclear (lamin B) markers was assessed by western blotting to confirm a correct fractionation (Figure 2A). Control and Lys treated nuclear lysates from three separate biological replicates were then subjected to 2-DE analysis. Three gels were processed simultaneously and analyzed using the ImageMaster TM 2D Platinum Software. Six protein spots of interest were successfully identified by MALDI-TOF-MS (Figure 2B,C and Table 1). These phosphoproteins are SKIV2L2 protein, sec-related protein D, T-complex protein 1 subunit delta, protein disulfide-isomerase A3 (PDIA3), coronin, actin-binding protein, 1C (CORO1C), mitogen-activated protein kinase 1 (MAPK1). mRNA expression levels of the six up-regulated proteins were assessed by RT-PCR. Results showed that in L-Lys-treated cells, the mRNA expression level of all tested gene were up-regulated (Figure 2D), which were comparable to the tendency of their protein expression changes. Additionally, protein expression of phospho-MAPK1 and MAPK1 were assessed by western blotting. The results showed that in L-Lys-treated cells, phospho-MAPK1 and MAPK1 expression level were up-regulated (Figure 2E,F, *p* < 0.05), which were consistent with the result of 2-DE.

**Table 1:** MS identification of up-regulated expressed protein spots in Lys-treated DCMECs.

Spot No.	Accession No.	Protein name	Gene name	MS/MS Scores	PI/MW (kDa)
1	IPI00839454	SKIV2L2 protein	SKIV2L2	143	5.94/118474.5
2	IPI00692563	sec-related protein D	SEC24D	162	6.56/113982.8
3	IPI00841695	T-complex protein 1 subunit delta	CCT4	367	7.52/58865.2
4	IPI00689325	protein disulfide-isomerase A3	PDIA3	182	6.23/57293
5	IPI00692453	coronin, actin binding protein, 1C	CORO1C	550	6.3/53776.2
6	IPI00713672	mitogen-activated protein kinase 1	MAPK1	668	6.5/41748.4

### 2.3. MAPK1 Up-Regulated Expression of Lactation Relative Proteins

**Figure 3:**
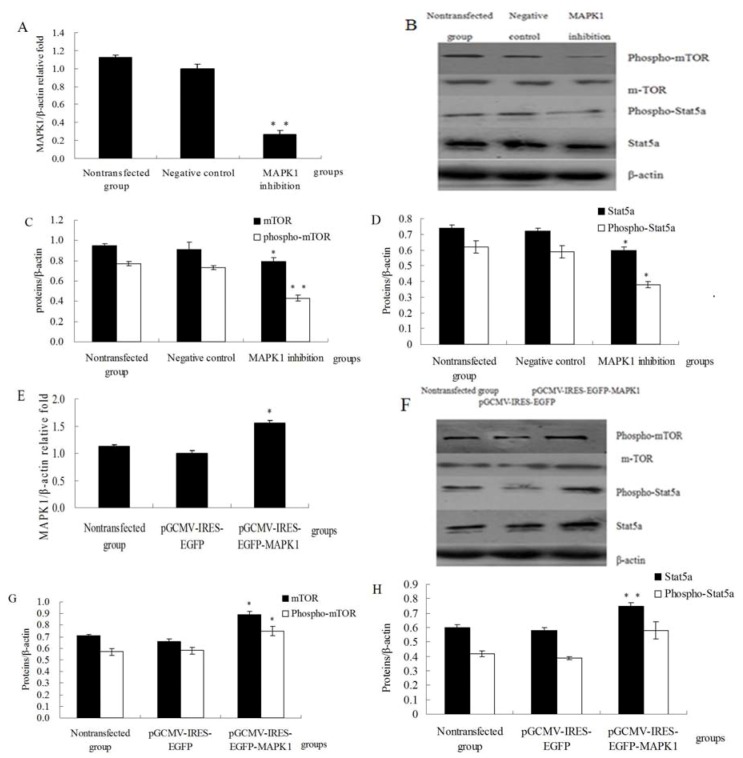
MAPK1up-regulated expression of Stat5a and mTOR (**A**) Relative mRNA expression of MAPK1 was determined by qRT-PCR after MAPK1 inhibition; (**B**) Western blotting results of phospho-Stat5a, Stat5a, phospho-mTOR, mTOR and β-actin after MAPK1 inhibition; (**C**) Results of gray scale scan of phospho-mTOR, mTOR/β-actin relative fold by western blotting after MAPK1 inhibition; (**D**) Results of gray scale scan of phospho-Stat5a, Stat5a/ β-actin relative fold by western blotting after MAPK1 inhibition; (**E**) Relative mRNA expression of MAPK1 was determined by qRT-PCR after over-expression of MAPK1; (**F**) Western blotting results of phospho-Stat5a, Stat5a, phospho-mTOR, mTOR and β-actin after over-expression of MAPK1; (**G**) Results of gray scale scan of phospho-mTOR, mTOR/β-actin relative fold by western blotting after over-expression of MAPK1; (**H**) Results of gray scale scan of phospho-Stat5a, Stat5a/β-actin relative fold by western blotting after over-expression of MAPK1. Values are means ± SE (n = 3 per group, biological replicates). * and ** indicate significant differences from values obtained in the negative control group or the pGCMV-IRES-EGFP empty vector group at levels of *p* < 0.05 and *p* < 0.01, respectively.

qRT-PCR was used to investigate mRNA expression of MAPK1. MAPK1 was downregulated in MAPK1 inhibition group compared with negative control group (Figure 3A, *p* < 0.01). As shown in Figure 3B, C and D, MAPK1 inhibition decreased the expression of phospho-mTOR (*p* < 0.01) and phospho-Stat5a (*p* < 0.05), and also mTOR and Stat5a (*p* < 0.05). MAPK1 was upregulated in the pGCMV-IRES-EGFP-MAPK1 group compared with the pGCMV-IRES-EGFP empty vector group (Figure 3E, *p* < 0.05). In Figure 3F–H, over-expression of MAPK1 increased the expression of phospho-mTOR (*p* < 0.05) and phospho-Stat5a (*p* > 0.05), and also mTOR (*p* < 0.05) and Stat5a (*p* < 0.01). These data indicate that MAPK1 may induce protein synthesis through the regulation of phospho-mTOR and phospho-Stat5 in DCMECs.

### 2.4. Discussion

2-DE has a special capacity to detect proteins of low abundance, such as regulatory proteins and transcription factors. The six up-regulated expressed phosphoproteins found in our proteomic analysis included MAPK1, SKIV2L2, Sec24D, Cct4, PDIA3, CORO1C. These proteins are involved in DNA transcription, mRNA translation, cell cycle, cell motility, and morphological processes. For the purposes of this study, those proteins involved in transcriptional and translational control were of particular interest. L-Lys is a necessary building block for all proteins in the body, can act as a signal molecule to activate MAPK1. In our experiment, over-expression and siRNA inhibition of MAPK1 showed that MAPK1 increased the expression and phosphorylation of mTOR and Stat5a. The MAP kinase is a very important signaling pathway in mammalian cells, activation of MAP kinase pathway result in a lot of cytokines releasing. MAPK1 is a proline-directed serine/threonine protein kinases that is activated in response to many extracellular stimulis such as amino acids. Raptor is a substrate for MAPK1, which phosphorylates Raptor to promote a Ras-dependent activation of mTORC1 [7]. The MAPK pathway could influence STAT5a functional capacity. In the mammary gland, Stat5a is well recognized for its function in lobuloalveolar development and milk protein expression [8]. There are some evidences that MAPK1 can enhance protein synthesis [9,10], and this is the first report that MAPK1 promotes milk protein synthesis in DCMECs. Up to date, the mechanism and function of MAPK1 in protein synthesis is not clear and need more research.

In this study, we found that L-Lys could promote the proliferation and casein synthesis of DCMECs. The ability of mammary glands to produce milk is determined by the number of cells and their level of activity [11,12], while β-casein, a major milk protein, also reveals the secretory ability of DCMECs. DMEM/F12 media is supplemented with L-Lys (0.499 mM), the level of L-Lys in DMEM/F12 media is a sufficient amount needed for normal cellular processes. The concentration of L-Lys for optimal DCMECs lactation was selected, and cells were stimulated with L-Lys (1.2 mmol L^−1^) for 2-DE analysis. We use this normal *vs.* stimulated model to select proteins involved in regulation of milk protein synthesis, comparing with starved vs restimulated model, this method could show fewer but more important proteins for analysis. An 5% increase in cell viability and 20% increase in the level of β-casein secreted in the media was detected upon L-Lys treatment. In our other previous experiment (data not reported), adding L-Met (0.6 mmol L^−1^) to DCMECs media lead to a ~5% effect on cell viability and near 30% increase in the level of β-casein secreted in the media, and these observations are consistent with L-Lys treatment described here. Appuhamy *et al.* showed that L-Lys stimulates the activity of S6K and affect mTOR-mediated cellular signals in MAC-T cells [13]. The well-characterized signaling pathway regulated by amino acids is the pathway of the mammalian target of rapamycin (mTOR) [14]. mTOR is a nutrient-sensing kinase that integrates the inputs from amino acids, growth factors, and the energy status of the cell [15]. Some studies showed that amino acid restriction suppressed the activity of mTOR in ATDC5 cells [16]. Together, Lys is essential donors of nitrogen in synthesis of milk protein, and also serves as signaling molecule to participate in the signal transduction pathway of milk synthesis. Nutrients affect protein translation in bovine mammary glands, but the molecular mechanism controlling milk protein synthesis is not clearly understood.

The time points of Lys treatment were selected according to previous reports [17,18]. The optimal incubation time with Lys was 24 h, at this time, the β-casein content got to the maximum (Figure 2). In the other hand, at 48 h and 72 h after adding Lys, the Lys concentration in the medium perhaps dropped down due to its utilization, thus we could not observe significant difference of the level of β-casein between control group and Lys group at these time points. Taking into account that phosphorylation of proteins of cell signal transduction pathway was correlated with milk synthesis, we speculated that phosphorylation of proteins for milk synthesis also got to the maximum.

Sec24D is subunit of COPII, which involves in transport of proteins. New synthesized milk proteins are transported through Sec24D mediated the secretory pathway in DCMECs [19,20,21]. Cct4 participates in the folding of newly synthesized polypeptides, including actin, tubulin, and several cell cycle regulators; and chaperonin Cct is a novel physiological substrate for p90 ribosomal S6 kinase [22]. Therefore, Cct plays an important role in maintaining the correct organization of DCMECs, and may mediates S6K related signal transduction pathway. Earlier work showed that Stat5 pathway is important for milk quality and milk protein synthesis [23]. In this work, protein MAPK1 was screened by comparative proteomics. Pircher *et al.* reported that MAPK interacts with Stat5a [24]. Western blot verified that phospho-mTOR and phospho-Stat5 was modulated by MAPK1. We also speculate that SKIV2L2 may be involved in pre-mRNA splicing for milk synthesis, PDIA3 has been reported to interact with STAT3-DNA complexes and regulate STAT3-dependent genes [25,26]. Moreover, PDIA3 is involved in the assembly of mTORC1 and positively regulates mTORC1 signaling [27]. PDIA3 might interact with the mTORC1 and STATs to affect milk protein synthesis, and phosphorylation of CORO1C might up-regulate milk protein synthesis through the PKC and PI3K signal pathway [28]. This study gives good avenues for further research on Lys regulation of milk synthesis, however, its precise mechnism still needs for further study

## 3. Experimental

### 3.1. Cell Preparation and Treatments

DCMECs from three biological replicates were provided by our laboratory. These cells were cultured according to the previous report [29]. DCMECs were grown in Dulbecco Modified Eagle Medium: Nutrient Mixture 12 (DMEM: F12) containing 10% fetal bovine serum (FBS), insulin (bovine, 5 μg mL^−1^), hydrocortisone (1 μg mL^−1^, Sigma), penicillin (100 U mL^−1^), and streptomycin (0.1 mg mL^−1^). For experimental assays, DCMECs in the logarithmic growth phase were plated at 3 × 10^4^ cells cm^−2^. Before applying the treatments, the medium was replaced with DMEM: F12 containing insulin (5 μg mL^−1^), hydrocortisone (1 μg mL^−1^), and prolactin (ovine, 3 μg mL^−1^) without FBS. To detect the dosage effect of L-Lys, cells were stimulated by adding L-Lys (0.3, 0.6, 1.2, 2.4, 4.8 mmol L^−1^) and were collected at 24 h as described previously [30]. To evaluate the effect of different treatments, cells were stimulated by adding L-Lys (1.2 mmol L^−1^), harvested after treatments at 0, 12, 24, 36, 48, and 72 h, respectively. Three parallel groups were prepared for each treatment and control groups were replaced with DMEM: F12 containing insulin (5 μg mL^−1^), hydrocortisone (1 μg mL^−1^) and prolactin (ovine, 3 μg mL^−1^) without FBS, also cultured at the same points.

### 3.2. Cell Viability Assay and Reversed Phase High Performance Liquid Chromatography

Cell viability was determined with a CASY TT Analyser System (Schärfe System GmbH, Reutlingen, Germany) according to the manufacturer’s instructions. After calibration with dead and vital DCMECs cells, cursor positions were set to 11.75 to 50.00 μm (evaluation cursor) and 7.63 to 50.00 μm (standardization cursor). Cells were trypsinized. The cells diluted with CASY electrolyte solution (1:100) was examined using CASY-TT, 100 μL aliquots were analyzed in triplicate.

Effect of length of incubation time with Lys on levels of β-casein secreted into media were measured using reversed phase high performance liquid chromatography (RP-HPLC, LC-10AT HPLC, Shimadzu, Tokyo, Japan) as described previously [31]. TSK-gel G3000PWXL (Tosoh, Tokyo, Japan) was used and the mobile phase was ultrapure water. The flow rate was 0.8 mL/min and the analytical wavelength was 280 nm. 

### 3.3. Nuclear Protein Extraction and Phosphoprotein Enrichment by Affinity Chromatography

Phosphate buffer could not be used throughout the procedure because phosphate ions interfered with subsequent phosphoprotein affinity chromatography. Cells were washed twice with cold TBS, and the nuclear proteins were extracted using the NE-PER kit (Pierce, Rockford, IL, USA).

Phosphoprotein-enriched fraction from nuclear proteins was obtained by affinity chromatography with the phosphoprotein purification kit (QIAGEN, Hilden, Germany), following the instructions of the manufacturer. Benzonase was used at twice the recommended concentration because of the high amount of nucleic acids present in the cell nucleus. For each preparation 2.5 mg of nuclear proteins was used. After running the column, protein-containing fractions were desalted and concentrated with Nanosep 10K ultrafiltration spin columns (Pall Corp., Port Washington, NY, USA).

### 3.4. 2-DE and Identification of Proteins by MALDI-TOF-TOF Peptide Mass Fingerprinting

2-DE and MALDI-TOF-TOF peptide mass fingerprinting were carried out as previously described [32].

### 3.5. RNA Extraction and Quantitative Real-Time PCR

Total RNA from primary cultured DCMECs was extracted using Trizol reagent (Invitrogen, Carlsbad, CA, USA), then further purified by phenol/chloroform extraction. Subsequently, the purified RNA was treated with DNase I (QIAGEN, Valencia, CA, USA). Total RNA (1 μg) sample was transcribed into cDNA using Thermoscript reverse transcriptase (TaKaRa, Dalian, China) according to the manufacturer’s protocol. RT-PCR and analysis were performed in a total volume of 20 μL using 96-well microwell plates and an ABI PRISM 7300 RT-PCR System (Applied Biosystems, Foster City, CA, USA). β-actin was used as the reference gene. The oligonucleotide sequences of forward primers and reverse primers for these genes were as follows: SKIV2L2: sense 5'-AAGTTATGCGAGAAGT TGC-3', antisense 5'-TGCTGTAATGGAGTAGGC-3'; SEC24D: sense 5'-TGATGTTCCACCATTCTA TTTC-3', antisense 5'-GGGTTGGGAGGCTTATTC-3'; CCT4: sense 5'-TTGTGAGTTGGTGGAA GG-3', antisense 5'-TTTGGGAGCAGATAAGCA-3'; PDIA3 sense 5'-AGCCAGCAACTTGAGGGAT AA-3', antisense 5'-TCCCACTGGTCATTTTCTGTTCT-3'; CORO1C: sense 5'-GCCATAATCATAG AAGCG-3', antisense 5'-CTCAGTCAGGGAAAGGGT-3'; MAPK1: sense 5'-GTCGCCATCAAGAA AATCAGC-3', antisense 5'-GGAAGGTTTGAGGTCAC GGT-3'; All RT-PCR reactions were performed at 95 °C for 10 s, followed by 40 cycles at 95 °C for 5 s, 60 °C for 31 s by using two-step RT-PCR. RT-PCR analysis was performed by the ∆∆CT method [33].

### 3.6. Western Blot Analysis

Briefly, cells in different treatments were washed twice with ice-cold PBS before scraping on ice with lysis buffer (20 mmol L^−1^ Tris-HCl, pH 7.5; 150 mmol L^−1^ NaCl; 2.5 mmol L^−1^ sodium pyrophosphate; 1 mmol L^−1^ sodium β-glycerophosphate; 5 mmol L^−1^ NaF, 1 mmol L^−1^ Na_3_VO_4_, 1 mmol L^−1^ phenylmethylsulfonyl fluoride, 1% Triton X-100, and 1 tablet of protease inhibitor mixture per 40 mL of lysis buffer). Cellular debris was removed by centrifugation (14,000 × g for 15 min at 4 °C). For SDS-PAGE, total cell lysate containing about 30 μg protein was separated on 10% SDS-PAGE gel and transferred onto nitrocellulose membranes (Bio-RAD, Shanghai, China). After blocking, the blots were probed with primary antibodies [1:200], rabbit polyclonal antibodies for MAPK1, phospho-MAPK1, Stat5a, phospho-Stat5a, mTOR, phospho-mTOR, β-tubulin and β-actin (Santa Cruz), mouse polyclonal antibody for lamin B (Santa Cruz); followed by a second incubation with secondary antibodies [1:1,000] conjugated to HRP (ZSGB-BIO, Beijing, China). The chemiluminescence detection of HRP-conjugated secondary antibodies was performed using Super ECL plus (ApplyGEN, Beijing, China). Each experiment was repeated three times.

### 3.7. Small Interfering RNA Transfection

Small interfering RNA (transfection MAPK1 inhibitor) and negative control RNA-oligonucleotides were purchased from ShangHai GenePharma. DCMECs were either transfected with the MAPK1 inhibitor or negative control using Lipofectamine^TM^ 2000 (LF2000) according to the manufacturer’s protocol (Invitrogen), and nontransfected cells were also conducted as controls in the same way of transfection. Briefly, DCMECs were grown in 6-well plates overnight, for cells of each well to be transfected, 1 μg of DNA and 2 μL LF2000 were diluted into 200 mL OPTI-MEM I medium, respectively. Once the LF2000 had been diluted, it was incubated with the DNA at room temperature for 20 min to allow the DNA-LF2000 complex to form, and then the complex was directly added to each well containing cells. Cells were incubated at 37 °C for 24 h. The cells were then collected for verification of MAPK1 knockdown by qRT-PCR and phospho-mTOR, mTOR, phospho-Stat5a and Stat5a expression by Western blot assay, respectively.

### 3.8. Construction of pGCMV-IRES-EGFP-MAPK1 and Transfection

The full-length of MAPK1 fragment was amplified by PCR, and primers designed with particular restriction enzyme sites were as follows. Forward primer 5'-CCCTCGAGATGGCGGCGGCGGCGGC GGCGG-3' (*Xho* I) and reverse primer 5'-CCCAAGCTTTTAAGATCGGTATCCCGGCTGG-3' (*Hin*d III). PCR fragment was subcloned into TA cloning vector (Promega) and completely sequenced. The full length of MAPK1was digested with *Xho* I and *Hin*d III, and then subcloned into pGCMV-IRES-EGFP vector (GenePharma). All constructs were subjected to sequencing for verification.

DCMECs were transfected with the pGCMV-IRES-EGFP vector and pGCMV-IRES-EGFP-MAPK1 vector using Lipofectamine^TM^ 2000 (LF2000) according to the manufacturer’s protocol (Invitrogen). Briefly, 1 × 10^6^ cells were seeded into an individual well of a 6-well plate. For cells of each well to be transfected, 1 μg of DNA and 2.5 μL LF2000 were diluted into 200 mL OPTI-MEM I medium, respectively. Once the LF2000 had been diluted, it was incubated with the DNA at room temperature for 20 min to allow the DNA-LF2000 complex to form, and then the complex was directly added to each well containing cells. Cells were incubated at 37 °C for 24 h. The cells were then collected for verification of MAPK1 overexpression by qRT-PCR and phospho-mTOR, mTOR, phospho-Stat5a and Stat5a expression by western blot assay, respectively.

### 3.9. Statistical Analysis

Results were reported as mean ± SE. Data statistics and individual differences among groups were analyzed using t-test by Sigma Plot 9.0. A value of *p* < 0.05 was considered significant differences and *p* < 0.01 was considered extremely significant differences.

## 4. Conclusions

Proteomic and functional analyses reveal MAPK1 regulates milk protein synthesis, though Stat5 and mTOR pathway in DCMECs.
